# 

**Published:** 1886-10-02

**Authors:** 


### The Hospital

*An Institution, Family, and Parochial Journal of Hospitals, Asylums, and all Agencies for the Care of the Sick, Criticism and News.*

(The Journal of the Hospitals Association.)

EDITED BY

HENRY C. BURDETT,

AUTHOR OF "HOSPITALS AND THE STATE"; "PAY HOSPITALS OF THE WORLD"; "COTTAGE HOSPITALS, GENERAL, FEVER, AND CONVALESCENT, WITH FIFTY BEDS AND UNDER: THEIR ORGANISATION, MANAGEMENT, AND WORK"; ETC., ETC.

VOL. I. FOR 1887.

(1st October 1886 to 31st March 1887.)

SURGEON GEN'L'S OFFICE LIBRARY 108921

LONDON:
PRINTED AND PUBLISHED BY WHITING & CO.,
30 & 32, SARDINIA STREET, LINCOLN'S INN FIELDS, W.C.

W1
H069X

### INDEX TO VOL. I., 1887.

Aberdeen Royal Infirmary, 94, 181
Abingdon, New Cottage Hospital for, 58
Accident, an, 42
Affairs at the Western Asylum, 94
After-care Association and H.R.H. Princess Christian, 162
Alcohol at the London Hospital, 41
Alcohol at certain General and Special Metropolitan Hospitals, 75
Amalgamation in Sunderland, 42
Ambassador, an unconscious, 28
Ambulance Association, The St. John's, 146
America and its charities, 299
An ambulance at work, 366
An appeal, 298
Ancient Order of Foresters' Convalescent Home, 233
Ancoats Hospital, the, 235
Animal Life, Incidents, by F. O. Morris, 117, 221, 290

**Annotations:**
- Advertising doctors and secret remedies, 62
- Alcohol a food? Is, 285
- Analysts on their defence, the, 165
- Annuities for a term of years, 405
- Asylums Board and clinical teaching, the, 115
- Aurard's incubator, 371
- Battle of St. Alban's, the, 303
- Books and periodicals, 149
- Cabs, infected, 98
- Chemists and druggists, 405
- Commission on Hospitals, a Royal, 285
- Cottage Hospitals, 131
- Coventry's care for its sick children, 184
- Dangerous reticence, 45
- Dead Hand, the, 27
- Dispensers and Hospitals, 62
- Drinks wanted, 389
- Eight years an out-patient, 149
- Exhibition of competition plans, 79
- Faith healing, 439
- Fever Hospitals in towns, 27
- First medical general election, the, 184
- Flogging in Board Schools, 165
- Glasgow, a third Hospital for, 353
- Guy's, the Alleged Inhumanity at, 434
- Home and the Hospital at Bristol, the, 321
- Homœopathy, Sir James Picton on, 335
- Hope for the hopeless, 45
- Hydrophobia, Society for its Prevention, 165
- Incurable and Chronic, *v.* Acute Diseases, 79
- Individual Contribution, and the Hospital Sunday, 115
- Just Complaint, a, 62
- Lady Doctors and Nurses, 131
- Libel on Hospital Sunday, a, 321
- Lodging House Keepers and their consciences, 98
- Lunatics and Doctors, 149
- Manson, Miss, and Trained Nurses, 389
- Medical Practice Humbug? Is, 335
- Metropolitan Local Press, the, 62
- Milk, cheap 27
- Nurses at Play, 353
- Newcastle leads the Van, 405
- Oban, a Hospital for, 201
- Out-Patient Question, 79
- Pay System, and the Hospitals and Provident Dispensaries, 115
- Physician? What is a, 421
- Practices, Hospital Sunday, to be avoided, 115
- Proposal to change Hospital Sunday, the 335
- Provident Dispensaries, 371
- Rates and Hospitals, 45
- Residential Accommodation for Medical Students, 98
- Responsible? Who is, 149

**Annotations**—*continued*
- Sane woman in a Lunatic Asylum, a, 421
- *Spectator* and Vivisection, the, 303
- Teeth at the General and Special Hospitals, 131
- Teeth and their Guardians, 421
- Temperance and Total Abstinence, 201
- Tenders, low, 27
- Town *v.* Country, 371
- Waking up at the Dorset County Hospital, 353
- Wanted £50,000, 131
- Week Movement, the Hospitals, 201
- Who stands sponsor for the matron, 389
- Word puzzles and gambling, 285

Amusements with profit, 324, 340, 358, 377, 427
Appeals, 181, 232
Appliances, sick-room, recent novelties, 144
Architects, Hospital, 146
Architects, Successful, 42
Asylum attendants for the insane, good, 62
Ashburton and Buckfastleigh Cottage Hospital, the, 403

Ball in Aid of the Italian Hospital, 300
Balls, Hospital and Infirmary, 350
Bath Hospital, Harrogate, 42
Bazaars, 198, 216
Bazaar and Fancy Fair at the Rink, Dublin, 216
Beau Site Convalescent Home, the, 350
Behnke, Mr. Emil, 78
Belfast Royal Hospital, 8
Bethnal Green Working Men's Society, 368
Bequests, 162, 216
Bequests to Medical Charities, 332
Birmingham General Hospital, the, 161, 403
Blind gentleman, an elderly, 28
Books, good and otherwise, 187, 289
Bournemouth, Proposed Hospital for, 112
Box Collections, 332
Brackley Cottage Hospital, 128
Bristol Children's Hospital, 418
Bristol Dispensary, the, 318
Bristol Eye Hospital, the enlargement of, 181
British Hospital at Port Said, the, 386
Bristol, Victoria Maternity Hospital at, 232
Brompton Cancer Hospital, the, 436
Brompton Hospital and Trained Nurses, 162
Brompton Hospital Musical Entertainments, 128
Burdett, Henry C., "Hospitals; the Sick Poor and all England," by,
Burdett-Coutts, Baroness, and the Great Northern Central Hospital, 418
Burnley Hospital, the, 42, 402
Burnley Mills and Workshops, the, 216

Camberwell Infirmary, the, 248
Cambridge, H.R.H. the Duke of, 94
——— and the German Hospital, 318
——— Importance of Maintaining Medical Charities, 106
Cancer Hospital, the, 368
Carrington, Dr. R. E., the late, 436
Champagne at the Eastern Hospital, 128
Chard and the Jubilee, 264
Charm and beauty of flowers, the, 47
Charing Cross Hospital, 24, 268
Charing Cross Hospital, Entertainment in aid of, 232
Chats about Medical Museums, 300, 442
Chelmsford, Lord, and the Convalescent Dinner Society, 318
Chelsea Hospital for Women, Fancy Dress Ball in aid of, 232
Chichester Infirmary, Improvements at the, 162
Child's Epistle, a, 350
Child-torture in Christian England, 257
Children's Hospitals in England, 136
Children, the East London Hospital for,
Children's Hospital at Paddington Green, the, 146
Children's Hospitals, 418
Children's Ministering Angels League, 235
Children's Corner, the, 15, 30, 50, 66, 84, 102, 137, 188, 207, 272, 291, 309, 340, 359, 376, 427
Children's ward, for the, 204, 383, 401, 415
Christ and the flowers, 47
Christmas Day at the Oldham Infirmary, 248
Christmas Trees, more, 282
Christmas Day in a London Hospital, 252
Christmas Festival, 251, 269
Christmas at the "Foundling," 275
Christian, H.R.H. Princess, and After-Care Association, 162
Church Parades, 76
Clark, Sir Andrew, Bart., M.D., F.R.S., "Hospital Co-operation and Mutual Help," by, 142
City Orthopædic Hospital, the, 232
Classes and Masses, by the Ven. Archdeacon Farrar, D.D., 193, 219
Clinical Assistants, 300
Clothing and health, 307
Collections, Hospital, 216
Collie, Dr., 24
——— on Enteric Fever, 76
Common accidents of every-day life, 288, 322
Competition among Doctors, 232
Competition Item, a, 58
Competition Prizes for Orphanage Designs, 181
Concert at St. John's Hospital, 300
Consumption Hospital for St. Leonards, the new, 332
Convalescent Hospitals, 8
Coope, Mr. O. E., the Late, 181
Corps of Volunteer Female Nurses, 8
Coster's Customer, the, or how I earned £20, by an Amateur Butler, 363
Cottage Hospitals, 76
Country Physicians, 121
Cremation Society of England, 76
Curious Hospital Incident, 418
Curious Mottoes, 282

**Difficult Cases:**
- Acute mania, 439
- An elderly blind gentleman, 28
- An intractable child, 14
- A poor half-witted girl, 256
- Cure of epileptics, 83
- Creeping paralysis in a lady, 187
- Employment for poor half-witted girls, 285
- Hopeless, Offensive and Incurable, 339
- Paralysis, a case of, 152
- Spinal curvature, a case of, 117

Darlington Hospital Scandal, the, 94
Deaf mutes and consanguineous marriages, 353
Decoration and emulation, 111
Derby, The Right Hon. the Earl of, "The Hospital Problem," by, 38
Derby, the Earl of, at Manchester, 42
Devonport, the Royal Albert Hospital, 216
Devonshire Hospital, Buxton, 42
Devonshire Hospital, Buxton, and Dr. Hazlerigg, 42
Dictionaries for Hospitals, 216
Difficulties of Covent Garden, 298
Digestion and dyspepsia, 354
Digestion, disorders of, 223, 308, 409
Discharged, by George Fleming, 4, 19, 36, 53, 71, 88
Disinfecting Linen, the best Tank for, 331
District nurse and her patients, 197
Doctor's workshops—poor-law infirmaries, 193
Doctors described by nurses and patients, 139
Dolly in the Hospital, 118

Donations, 232, 248, 300
Donation to the Longmore Hospital by the Queen, 128
Donations to Medical Charities, 300
Donations and Legacies to Medical Charities, 368, 386, 402, 418
Donations to Medical Charities, 436
Dramatic Performance in Aid of the Cancer Hospital, 332
Driffield Cottage Hospital, the, 162
Dublin, New Hospital for, 216
Dudley Dispensary, the, 94
Durham County Hospital, 58
Durham, Bishop of, and the Newcastle Infirmary, 76
Dyce-Davidson, Prof., 76

East London Hospital for Children, 418
East London Nursing Society, 418
Editor's Letter Box, 13, 26, 48, 82, 101, 136, 161, 222, 255, 280, 331, 375, 393
Elephant Man, the, 264
Employment for Epileptics, 136
Employment for Epileptics, by Miss Nina Paget, 347
Entertainment at the Windsor Royal Infirmary, 332
Epileptics, Cure of, 83
Essex Convalescent Home, the, 368
Eye Hospital for Cardiff, new, 128

Fancy bazaars in aid of the Hospitals, 318
Farrar, the Ven. Archdeacon, D.D., Classes and Masses, by, 193, 219
Farthing Dinners, by a Young Hand, 384
Fever and Small-pox Hospitals, 128
Fever cases in the Metropolis, 94
Few November Flowers, a, 136
Financial depression at the Metropolitan Hospitals to-day and ten years ago, 195
Fishermen's Cottage Hospital for Yarmouth, a, 282
Fleming Memorial Hospital, the, 216
Flowers, Ferns, and Ward Decorations, 28, 47, 65, 100, 111, 145, 186, 239, 298
Flowers for Hospitals and Schools, 255
Flowers as symbols, 65
Flowers, how to pack them, 145, 186
Flowers in every-day life, 28
Flower Services, 145
Four Christmas Days in Hospital, 248
Friendly Societies and the Hospitals, 94
Fruit and linen for Hospitals, wanted, 58
Fruit for Hospitals, 83

Gardens, 28
General enquiries, 14
General Hospital, the Birmingham, 161
German Hospital and the Duke of Cambridge, 318
Glasgow, the Lord Provost of, and the Hospitals, 386
Glasgow University and Medical School, 257
Giving flowers, 100
Good work done, and to be done, 69
Good work well finished, a, 259
Globe Parcel Delivery Co., the, 182
Grand Jubilee Doll Show, 282
Grateful Patient, a, 282
Gratitude of Hospital Patients, b a Practical Philanthropist, 237
Great Northern Central Hospital, the, 146, 232, 300
Groan of the willing horse, the, 45
Guest Hospital at Dudley, 162, 282
Gulson, Mrs. S. L., Founder of the Coventry Children's Hospital, 106
Guy's Hospital, 232; alleged inhumanity at, 434

Half-witted girls, poor, employment for, 285
Half-witted girl, a poor, 256
Halsbury, Lord, and National Hospital for Paralysed and Epileptic, 461
Hampstead Home Hospital, the, 402
Harvey interviewed, 138
Hazlerigg, Dr., and the Devonshire Hospital, Buxton, 42
Heanley, K. M., Manual of Urine Testing, by, 82
Heart diseases, a word of comfort, 365
He laid down his life, 313
Help at hand, brother, 14
Highgate Infirmary, 76
Homes on the Riviera, 101
Homes for Incurables; free payments *v.* the voting system, by Miss Lousia Twining, 281
Home Wanted, a, 26

Hospital alms-boxes, 436
Hospital anecdotes, 436
Hospital and Infirmary reports, 162
Hospital and Asylum superintendents, 8
Hospitals' Association, the, 208
Hospital collections, 216
Hospital dressers, 24
Hospital enlargement, 264, 436
Hospital imposture, 216
Hospital Saturday at New York, 318
Hospital Saturday Fund, 101, 368
Hospital smuggling, 248
Hospital Sunday in the Metropolis, 318
Hospital Sunday movement in Birmingham 112
Hospital students and their patients, 198
Hospitals, and Boys' and Girls' Schools, 280
HOSPITAL, THE, Elementary Schools and the Public, 48
Hospital revenues to-day and ten years' ago, 173
HOSPITAL, THE, Charity Box, 16, 30, 49, 66, 83, 102, 137, 188, 207
Hospitals' Association conversazoine, the, 126
Hospital promotion, a wag's experience, 122
Hospital Co-operation and Mutual Help, by Sir Andrew Clark, Bart., F.R.S., 142
Hospitals for lady probationers, 206
Hospital huts, 392
Hospital Matron, a, on Hospital Morals, 218
Hospital Problem, the, by the Rt. Hon. the Earl of Derby, 38
Hospital Sunday Fund, Metropolitan, 1886, 206
Hospital sister, alleged cruelty of, 123
Hospital celebration of the Queen's Jubilee, 247
Hospitals, the Sick Poor, and all England, by H. C. Burdett, 295
Hospital Administration, 5, 41, 75, 173, 195
Hospital Worthies, 11, 21, 106
How to enter the medical profession, 185
Howard, John, on registration fee payments, 112
Hull Infirmary, the, 331

Imposture, Hospital, 216
In a Hospital ward, 111
In- and out-patients' Hospital Diary, the, 16, 32, 67, 85, 103, 120, 138, 154, 170, 189, 208, 224, 240, 273, 292, 310, 443
Incubators for Infants, 393
India, Hospitals in, 368
Infectious *v.* Non-infectious Convalescents 393
Infectious fevers and diseases in London, 80, 354
Intractable Child, an, 14
Invalid Passengers on Board Ship, 82
Insane, reminiscences of the, 133, 167, 204, 336, 372
Isolation huts, an enquiry from America, 223
"Is it nothing to you, all ye that pass by?" 100

Jaffray Hospital, Madame Roze at the, 386
Jonah and the Whale, 409
Jubilee celebration and the hospitals, 402
Jubilee commemorations, 436
Jubilee Cottage Hospital for Melton, 162
Jubilee Hospital proposals, 368
Jubilee proposals, 248, 282
Jubilee memorials, 181

Kensington District Nursing Association, 128, 300
Kilmarnock Infirmary, gifts to, 146
Kyrle Society, the, 146, 181

Lancaster town, its Mayor and Hospital, 263
Latest of the water cures, 323
Lectures at the Midwives Institute, 332, 402
Leeds medical charities, 280
Legacies, large, 58
Legacies to the Brompton Consumption Hospital, 162
Legacies to the Manchester Hospitals, 198
Leicester Infirmary, meeting of governors, 282
Lincoln County Hospital, the, 282
Literary prizes, 102, 157
Llanelly Hospital, 128
London Dental Hospital, the, 436
London Hospital, the, 24
London Rheumatic Hospital, the, 24
London School of Medicine for Women, 58

London smoke, 111
Longmore Hospital, the Queen at, 121
Lord Mayor and the Marylebone Infirmary, the, 248
Lowther Lodge, entertainment at, 386
Lunatics in England and Wales, 8
Lying-in Hospital, the City of London, 402

Macclesfield Infirmary, the, 386
Maintenance of the Hospitals, Lord Meath on, 146
Maintenance of Medical Charities, H.R.H. the Duke of Cambridge on, 106
Management of the eye, ear, and throat, 255
Mania, Acute, 439
Marvellous calculations, 248
Maternity Hospital at Bristol, Queen Victoria, 232
Medical books and general readers, 105
Medical charities, 318
Medical classes at Aberdeen University, 146
Medical Museums, Chats about, 390, 442
Medicinal Properties of Woodhall Spa Waters, 331
Medicine bottles for Hospitals, 316
Meeting of delegates, 368
Meeting of the Hospitals Association, 402
Meeting of the governors of Leicester Infirmary, 282
Memorial at Leicester Infirmary, 318
Memorials, the Queen's Jubilee, 181
Mental excitement, 58
Mercers' Hospital, Dublin, 216
Mercier, Mrs., Presentation to, 216
Metropolitan Convalescent Institute, 318
Middlesex Hospital, 24
Midwives, the Training of, 331
Mildmay Mission Hospital, by a Young Hand, 349
Milk and Alcohol in Children's Hospitals, 101
Miller Hospital at Greenwich, 76
Mine Women, proposed Hospital for, 300
Mischievous mis-statements, 395
Miscellaneous items, 8, 24, 42, 58, 76, 94, 112, 402, 418, 128, 162, 181, 198, 216, 232, 248, 262, 282, 300, 318, 332, 350, 368, 386, 436
Mogg Memorial Hospital, the, 128
Mold Cottage Hospital, the, 436
Morley House, munificent donation to, 332
Morley, Samuel, 21
Morphio-mania, 325
Morris, F. O., Animal Life Incidents, by, 117, 221, 290
Munificent bequests, 112, 146
My ward, my home, 111

Nairn Hospital and the Sanitary Committee, 198
National Hospital for Paralysed and Epileptic, 222
National Hospital for Paralysed and Epileptic and Lord Halsbury, 368
National Pension Fund for Hospital officials and nurses, 406, 435
National uses of flowers, 65
Netherfield Road Hospital, the, 146
Newcastle Infirmary and the Bishop of Durham, 76
New Chapel for Dublin, 76
New Children's Hospital for Newcastle, 76
New Clinical Hospital for New York, 112
New Cottage Hospital for Ripon, 216
New Home for English and American girls in Berlin, 232
New Hospital for Dublin, 216
New Hospital at Hastings, 112
New Hospital for St. Alban's, 248
Newsvendor's legacy, a, 436
Noble Profession, a, 52, 92, 227, 315
Notes and News, 8, 24, 42, 58, 76, 94, 112, 128, 146, 162, 181, 198, 232, 248, 264, 282, 300, 318, 332, 350, 368, 386, 402, 418, 436
Norwich Hospital Sunday Fund collection, the, 264
Notices to correspondents, 1, 17, 50, 66, 84, 101, 119, 136, 153, 169, 207, 272, 291, 309, 340, 358, 377
Nottingham General Hospital, the, 112
Nurse farms, hospitals, and private nursing institutions, 413, 433
Nurse Marion's Romance, 431
Nurses' Recreations, 379
Nurses' Food, 48, 82, 101
Nurses' and Probationers' Food, 48, 82, 101
Nurses' Pension Funds, 375
Nurses' Remuneration and Pensions, 136

Nursing in Workhouse Infirmaries, 440
Nursing Institutions and Hospitals, 381
Nursing fevers in London, provision for, 139
Nursing the sick, a noble profession, 52, 92

Officers' Food and the Regulation of Meals, 13, 48
Opening of the Cardiff Eye Hospital, 282
Ophthalmic Hospital, the Royal London, 418
Opportunity for good, a great, 357
Ought Asylums to rank as Hospitals? 35
Our skin, and how to take care of it, 391
Out-patients, among the, by One of the Crowd, 178, 329
Out-patients' registration-fee system, by J. C. Steele, M.D., 97
Out-patients subscription boxes, 76
Overcrowding at Hospitals, 318

**Poetry:**
Blind, the, 13
Few November flowers, the, 81
Growing Old, 356
Hospital Ward, In a, 57
Help the Hospitals, 192
In the Children's Ward, 326
In the Night-time, 156
Modern Samaritan, the, 132
Patience, 430
Plea for the Dumb Animals, 93
Retrospect of Christmas, a, 376
The One Lord of Life, 294
Paget, Miss Nina, Employment for Epileptics, by, 347
Paralysis, Creeping, in a lady, 187
Paralysed and Epileptic, Lord Halsbury and the National Hospital for the, 368
Past, Present, and Future of the Hospitals, the, by Sir Sydney Waterlow, Bart., 143
Pauper Infirmaries, old-fashioned, 90
Pauper lunatics, increase of, 146
Pauperism, golden age of, 155
Payment of nurses, the, 350
Penzance Convalescent Home, 216
People's Want in North London, the, 283
Persistence not Panic, 429
Peterborough Infirmary, the, 368
Pianos for Hospitals, 282
Picton, Sir James, on Homœopathy, 335
Pictures in the wards, 58
Plans for Hospitals, 282
Poor-law Infirmaries, medical education and the people, 51
Pot plants for Hospitals, 239
Presentation of busts to the Manchester Royal Infirmary, 264
Presentation to Mrs. Mercier, 216
Presentation to the founders of the Stanley Hospital, 300
Prince Albert Victor at Burnley, 42
Prince's Cinderellas, the, 94
Prize competitions, 15, 30, 49, 65, 290, 309
Proposed Hospital for Southend, 248
Public and its Physic, the, 411

Queen's Jubilee, 171
Queen and the East End Hospitals, the, 402
"Queen's Bed, The", at the North London Hospital, 146
Questions and answers, 434
Quilter prizes, the, conditions, etc., 115, 134, 150, 187, 339, 355, 417

Registration-fee system, 73, 76
Registration-fee payments, John Howard on, 112
Relic of John Hunter, 330
Remedies, new and old, 231
Re-opening of the West London Hospital, 128
Reports, Hospital and Infirmary, 300
Retirement of Mrs. Rhan, 264
Retrospect of Christmas, a, 376
**Reviews and Notices:**
Convalescent Homes for Asylum Patients, 81
Handbook on Nursing, a, by Catherine Jane Wood, 82
Manual of Urine Testing, a, by K. M. Heanley, 82
Practical Medicine, by Sir James Sawyer, M.D., 118
Home Remedies, 118
Ambulance Lectures, 152
Concerning Medical Students, 153
Richmond Hospital, a Correction, 331
Rider, Dr., resignation of, 198
Roze, Madame, at the Jaffray Hospital, 386
Royal Hospital for Diseases of the Chest, 436
Royal Maternity Charity, the, 318

Salisbury, Marquis of, K.G., on Hospital Support, a Public Duty, 140
Salisbury's, Lord, Appeal for the Hospitals, 181
Santa Claus Garner, 212
Saturday, Hospital, at New York, 318
Saturday Fund, Hospital, 368
Sawyer, Sir James, "Practical Medicine," by, 118
Serious Question, a, 271
Sewage Question, ethics of, 155
Should "Sisters" and "Nurses", marry? 236
Sick-room walls, best cover for, 135
Sister Olive, by W. J. Eccott, 159, 175, 202, 214, 229
**Special Notices:**
HOSPITAL, THE and the *Globe*, Lord Salisbury's article, 180, 198
Journalistic enterprise extraordinary, 162
Steele, J. C., M.D., Out-patients' Registration Fee System, by, 97
St. Leonard's, new Consumption Hospital at, 332
St. Mary's Hospital, 24
Seamen's Hospital, the, 386
Sheffield School of Medicine, 76
Sherburn Hospital and the Charity Commissioners, 181
Sick Poor, the North London Association for the, 418
Sister Dora, 58
Sister Olive Question, the, 248
Smuggling, Hospital, etc., 248
Soho Hospital for Women and the Duke of Westminster, 264
Staff Nurses Promoted, 42
Statue of Sister Dora, 76
Stock Exchange Collection, 322
Story of Sister Olive, the, 256
Story of the Hospitals, the, by One of the Public, 9, 55, 109, 157, 210, 260, 296, 327, 397
Stratford Dispensary, 146
Stroud General Hospital, the, 386
Students, medical, 153
Students, medical, their schools and schoolmasters, 86
Students, Hospital, and their Patients, 198
Sturge, Mr., and the Alcohol Question, 5
St. Luke's Guild, 184
Subscription Boxes in Out-patient Departments, 101
Superintendents, Hospital and Asylum, 8
Sunday, Hospital in the Metropolis, 318
Sunday Movement in Birmingham, the Hospital, 112
Surgical Aid Society, 216

Taunton and Somerset Hospital, 94
Teeth, take care of your false, 149
Temporary Hospitals in Liverpool, 76
Thefts, Hospital Collecting box, 402

**Till the Doctor comes:**
Bathing and beds, 205
Diseases of children, 29, 64, 96, 134
Feeding, preservation of ice, 238
Fomentations, lotions, and bed sores, 356
Fainting fits, 426
General hints on nursing, 168
Poultices, 336
Practical hints for the sick-room, 269
Sick appliances for the household, 306
Total Abstinence and Common Sense, 161
To our readers, 1
Toys for Poor Children, 94
Trade Societies' Demonstrations, 94
Tradesmen's Presents, 216
Trained Nurses 24
Trained Nurses' Club, 112
Training School for Nurses, 8
Training of Midwives, 136, 169
Training for Cottage Hospital Matrons, 26
*Truth* Toy Fund, the, 166, 198
Twining, Miss Louisa, "Homes for Incurables; free payments *v.* the Voting System," by, 281

Ulcerated legs, their care and cure, 316
Umbrella for a rainy day, an, 416
Unexpected Donation, an, 368
Unite and co-operate, 343
Uniformity in Hospital Accounts, 375
University College Hospital, 436
Upton Lunatic Asylum, 112

Vacancies, 76, 94, 128, 146, 162, 181, 198, 216, 248, 264, 282, 300, 318, 332, 368, 386, 402, 418, 436
Vanderbilt, W. H., the late, 112
Volunteer Female Nurses, Corps of, 8

Wakley, Dr. Jas. Goodchild, 11
Wales, H.R.H. the Prince of, and Exhibition Boxes, 42
Waterlow, Sir Sydney, Bart., "Past, Present, and Future of the Hospitals," by, 143
Weather and the Hospitals, the, 282
Well done, 17
Wellington Dispensary and the Resignation of Dr. Rider, 198
Wesleyans and Hospital Sunday, the, 280
West Bromwich District Hospital, 58
West Ham, the proposed Hospital for, 58
West Ham, Hospital Sustenation Fund, 112
West London Hospital, the, 128, 198, 402
Westminster Hospital, 248
Westminster Hospital, Improvements at, 42
Westminster, the Duke of, and the Hospital for Women, Soho, 264
What all can do, 100
Who did it? by Miss Laura Bambridge, 243
Willie: a true story, by a Cottage Hospital Nurse, 150
Wood, Miss, on the Management of Infants and Children, 198
Worcester Ophthalmic Hospital, 368
Words of consolation, 7, 23, 40, 57, 74, 93, 110, 132, 104, 226, 242, 258, 276, 294, 312, 326, 344, 362, 380, 412, 430
Worse than useless, 145
Workhouse Infirmary, the modern, 124
Workhouse nursing, how not to do it, 338
Workhouse visiting and management, 317
Workers *v.* beggars, 311
Working Classes and the Hospitals, the, 112
Workington Infirmary, the, 181, 216
World of babies, a, 407
Worthington, Mr. the resignation of, 42
Wrexham Infirmary, the concert at, 162

THE HOSPITALS ASSOCIATION,
NORFOLK HOUSE, NORFOLK STREET, STRAND, W.C.
*President*—SIR ANDREW CLARK, Bart., M.D., F.R.S.
EVERY INSTITUTION REPRESENTED BY THE MEMBERS OF THE ASSOCIATION, AND ALL ITS MEMBERS, ARE SUBSCRIBERS TO "THE HOSPITAL."

### NOTICE TO CORRESPONDENTS.

*All correspondence must be written on one side of the paper only.*

*We cannot undertake to return MSS. not used.*

Communications, *whether intended for insertion or for private information, must be authenticated by the names and addresses of the writers, not necessarily for publication.*

Local papers *containing reports or news paragraphs* should be marked.

*It is especially requested that early intelligence of* local events of interest, *or which it may be thought desirable to bring under notice, may be sent direct to the Editor.*

*Communications respecting editorial matters should be addressed to the Editor, Norfolk House, Norfolk Street, Strand, London, W.C.*

### TERMS OF SUBSCRIPTION.

*Subscribers to* THE HOSPITAL *residing in London and the Suburbs will receive their Copies by the first post on* FRIDAY MORNING. *Copies for Country Subscribers are forwarded by the* DAY *Mail on Fridays. Terms, including postage, payable in advance:*

| | Weekly Parts. | Monthly Parts. |
|---|---|---|
| Twelve months | 0 6 6 | 0 7 6 |
| Six „ | 0 3 3 | 0 3 9 |
| Three „ | 0 1 8 | 0 1 11 |

*Post Office Orders should be made payable at the General Post Office, London, E.C., to* WHITING AND Co., 30 *and* 32, *Sardinia Street, Lincoln's Inn Fields, W.C. Cheques to be crossed "London and Joint Stock Bank, Limited," Chancery Lane Branch.*

Cases for Binding *the weekly or monthly parts of* THE HOSPITAL *in yearly vols. may be ordered of the Publishers.*

### BUSINESS COMMUNICATIONS AND ADVERTISEMENTS.

*All communications concerning business matters, non-delivery of* THE HOSPITAL, *etc., should be addressed to the Manager, at the publishing offices,* 30 *and* 32, *Sardinia Street, Lincoln's Inn Fields, London, W.C.*

Cheap advertisements *of the "Wanted" class, including those for situations, of Twenty-four Words or under, are charged* 1s. *an insertion, or three insertions for* 2s. 6d., *but must be paid for in advance. Each additional line of Eight Words is* 4d.

Employers requiring assistants *are charged for one insertion of Twenty-four Words or under,* 2s. 6d., *or* 6s. *for three insertions. Each additional line of Eight Words is* 1s.

Advertisements *for insertion in the weekly issue of* THE HOSPITAL must be delivered *at the publishing offices* not later than 11 a.m. on Wednesdays.

*Cheques and Post Office Orders to be made payable to* MESSRS. WHITING & CO. *Subscribers and Advertisers are requested* not to send stamps.

**The Hospital.**

An Institution, Family, and Congregational Journal of Hospitals, Asylums, and all Agencies for the Care of the Sick, Criticism and News.

SATURDAY, OCTOBER 2ND, 1886.

### TO OUR READERS.

### "THE HOSPITAL" PRIZE COMPETITIONS.

We offer for competition among our readers money prizes, classified under the subjoined heads. We are anxious that this feature of THE HOSPITAL shall prove of interest to all classes of readers, and the grouping of the competitions has been arranged solely with this end in view.

All competitors who send in papers must strictly abide by the following

### Conditions.

I. Every paper sent in must be clearly written, and one side only must be used. All competitors must mark at the head of their papers the number of their competition.

II. Each paper must be signed by the author with his or her real name and address A *nom de plume* may be added, if the writer does not desire to be referred to by us by his real name. In the case of all prize-winners, however, the real name and address will be published.

III. All communications relating to prize competitions should be addressed to "The Prize Editor, *The Hospital* Office, Norfolk House, Norfolk Street, Strand, W.C" Competitors are requested not to include in letters, etc., to the Prize Editor communications on matters of other business. All letters must be fully prepaid.

IV. The Prize Editor of *The Hospital* is the sole judge of the competitions, and his decision is in all cases final and without appeal.

**V. All competitions must be received at "The Hospital" Office by the dates stated, failing which they cannot be dealt with. Every Reply must be accompanied by the Prize Competition "Coupon" which appears on another page.**

VI. The Prize Editor reserves the right to publish any paper sent in, whether it receives a prize or not. He does not hold himself responsible for any MSS. sent, nor can he undertake to return rejected competitions.

VII. No competition must exceed two columns in length, except the Story competition, which must not exceed six columns.

### No. 1.—The One Guinea Prize.

A prize of *One Guinea* will be offered every fortnight to all readers of *The Hospital* for the best *Story* or narrative based upon hospital, asylum, or student life, or upon any incident connected with the professions of medicine or nursing. The Prize Competition will be published in *The Hospital.*

Competitions must be received by Saturday, 16th October. Envelopes to be marked in left-hand corner, "Story."

### No. 2.—The Half-Guinea Prize.

A prize of *Half-a-Guinea* is offered fortnightly to in and out-patients for the best *Anecdote* or *Personal Reminiscence* of any interesting circumstance which may have come under their observation while at or attending any hospital.

Competitions must be received by Saturday, 16th October. Envelopes to be marked in left-hand corner, "Anecdote" or "Reminiscence."

### No. 3.—The Five Shilling Prize.

A Prize of *Five Shillings* is offered weekly to all readers of *The Hospital* for the best *Item of News* suitable for publication in this Journal.

Competitions must be received by Saturday, 16th October. Envelopes to be marked in left-hand corner, "News."

### No. 4.—The Nine Pounds Quarterly Prizes.

Prizes of *Five, Three,* and *One Pound* will be awarded on 2nd January, 1887, to the three persons who shall have obtained during the months of October, November, and December, 1886, the largest number of subscribers to *The Hospital.* Order Forms, for subscribers to fill up and sign, may be obtained on application to the publishers, 30 and 32, Sardinia Street, Lincoln's Inn Fields, London, W.C. The Prize Editor must be informed by competitors from time to time of the names and addresses of new subscribers they procure. Names will be received up to and including Saturday, 18th December, 1886.

### THE CHILDREN'S CORNER.

In arranging the lines on which we propose to conduct this Journal, we feel we ought not to neglect the little ones, whom we hope to reckon among our readers, and who will, we trust, become our correspondents. We have, therefore, reserved a corner for our juvenile readers, some of whom, when they make our acquaintance, will doubtless be within the walls of our hospitals. But whether they are sick or well, it is our intention to make this corner one of interest and pleasure to them. The solution of our pastimes will, perhaps, divert the weary hours of convalescence. Riddles, charades, acrostics, buried cities and the like, will form a weekly *mélange*, the interest of which we shall stimulate by rewards.

### OUR PASTIMES.

We commence to-day our first Children's Quarterly Competition. The prizes we offer are :—

| | |
|---|---|
| 1st ... ... ... | Thirty shillings. |
| 2nd ... ... ... | Twenty ,, |
| 3rd ... ... ... | Ten ,, |
| 4th ... ... ... | Five ,, |

which will be awarded to the four competitors who shall have sent in, during the thirteen weeks, the largest number of correct answers to our pastimes. Let us commence :

1. There is a certain word of four letters, from which, if you take away one letter, only one remains. Who can guess it ?

2. If a little girl desires to inform her music master that she is able to play her new piece, what disease will she in all probability unconsciously name ?

3. Here is a little sum in puzzle arithmetic Find the value of

$$\frac{100+50}{0} + \tfrac{2}{3} \text{ of } 10.$$

Tis thus the question tends,
And so, you know, it ends.

4. The first two letters of the word,
Denote my sex is male ;
But if you wish to add a third,
A female forth I sail ;
Add yet a fourth and I'm a he,
Add yet three more, then I'm a she.
What is the word ?

Answers, having the actual name and address (to which a pseudonym may be added for publication), must have "The Children's" Coupon attached (see advertisement pages), addressed to the "PUZZLE EDITOR, THE HOSPITAL, Norfolk House, Norfolk Street, Strand, W.C.", not later than Thursday, 7th October. Results will appear in our issue of 16th instant.

### THE HOSPITAL "CHARITY BOX."

OUR "Charity Box" is intended to afford a little practical help to the hospitals. Each week we shall propose a word for "anatomical dissection," and the first 150 competitors who extract the largest number of words from it will receive the following prizes :—

| | | |
|---|---|---|
| 1st | ... ... | One Guinea. |
| 2nd | ... ... | Half a Guinea. |
| 3rd | ... ... | Five Shilliings. |

The above Prizes will be repeated for every additional 150 competitors who may enter each week.

FIRST COMPETITION.

Compose the largest number of words you can from the name—

ABERNETHY.

*Observe:* Proper names, abbreviations, foreign words, and repetitions are barred.

Write only on one side of the paper, and tota your words.

Append your real name and address, and add, if desired, a pseudonym for publication.

All replies, addressed "Charity Box," THE HOSPITAL, Norfolk House, Norfolk Street, W.C., must arrive by Saturday, October 9th. Results will appear Saturday, October 16th.

*Every competitor is required to cut out and enclose with his list the "Charity Box" Coupon* (see advertisement pages), *and a shilling entrance fee.*

The proceeds resulting from each "Charity Box" competition will be funded until the end of December 1886. The total sum available for distribution, and the names of the hospitals selected for donations, will appear on Saturday, January 8, 1887. The distribution of the proceeds will be at the Editor's sole discretion.

### THE OUT-PATIENTS' HOSPITAL DIARY.

The following Table gives the terms of admission, the names of the physicians and surgeons, and the days and hours of their attendance upon the out-patients at all the general hospitals in the Metropolis. The same facts will be given about all the special hospitals next week, but to-day the account is limited to the arrangements made throughout London for seeing those who are suffering from general diseases and accidents.

| Hospitals and Terms of Admission. | Medical Officers, and Days and Hours of Attendance. | |
|---|---|---|
| | Physicians. | Surgeons. |
| CHARING CROSS HOSPITAL, West Strand, W.C.<br>Out-patients admitted without subscribers' letters first time; for continued attendance they must be obtained from the Secretary | Dr. Willcocks<br>„ Sangster<br>„ Abercrombie<br>„ A. Routh<br>„ Lubbock<br>„ Murray<br>Daily, 1.30 p.m. | Mr. Cantlie<br>„ J. H. Morgan<br>„ Stanley Boyd<br>Daily, 1.30 p.m. |
| FRENCH HOSPITAL, 10, Leicester Place, Leicester Sq., W.C.<br>Free to all foreigners speaking French | Dr. Vintras<br>Tu., F., 10 to 11 a.m. | M. De Méric<br>„ Keser.<br>M., W., Th., S., 10 to 11 a.m. |
| GERMAN HOSPITAL, Dalston Lane, E.<br>Free, or by Governor's letter | Dr. Tuchmann<br>„ Ludwig<br>„ Keller<br>„ Reihlen | *Women*, M., W., F., 2 p.m.<br>*Men*, Tu., Th., 2 p.m. |
| ——— Eastern Branch, 49, Mansell Street, Aldgate, E. | Dr. C. Harrer | Tu., F., 8 to 10 a.m. |
| ——— Western Branch, 259, Oxford Street, W. | Dr. M. Castaneda | M., T., 8 to 10 a.m. |
| GREAT NORTHERN CENTRAL HOSPITAL, Caledonian Road, N.<br>Free to all | Dr. Beevor<br>„ Beale<br>M., Tu., Th., 2 p.m.; S., 10 a.m. | Mr. Lockwood<br>„ G.H. Makins<br>M., Tu., Th., F., 2 p.m. |
| GUY'S HOSPITAL, St. Thomas's Street, S.E.<br>Admission free | Dr. Goodhart<br>„ Carrington<br>„ Hale White<br>M., W., Th., F., S., 12.30 p.m. | Mr. Lucas<br>„ Golding Bird<br>„ Jacobson<br>„ Symonds<br>M., W., Th., F., S., 12.30 p.m. |
| KING'S COLLEGE HOSPITAL, Portugal Street, W.C.<br>By Governors' letters; urgent cases free at all times | Dr. Ferrier<br>„ J. Curnow<br>„ N. I. C. Tirard<br>*Men*, Tu., Th., S., 1; *Women*, M., W., F., 1 p.m. | Mr. W. Rose<br>„ W. Cheyne<br>*Men*, Tu., Th., S., 1; *Women*, M., W., F., 1 |
| LONDON HOSPITAL, Whitechapel Road, E.<br>Admission free | Dr. Sansom<br>„ Ralfe<br>„ Turner<br>„ Warner<br>„ Gilbart Smith<br>Daily, 1.30 p.m. | Mr. Mansell-Moullin<br>„ Eve<br>„ Reeves<br>„ H. Fenwick<br>Daily, 1.30 p.m. |
| LONDON HOMŒOPATHIC HOSPITAL, Great Ormond Street, W.C.<br>By registration card from hospital dispenser | Dr. J. G. Blackley<br>„ J. H. Clarke<br>„ W. Epps<br>„ T. Anderson<br>„ Renner | Daily, 2.30 to 3 p.m. |
| LONDON TEMPERANCE HOSPITAL, Hampstead Road, N.W.<br>For treatment without alcohol | .... | Mr. G. C. Wilkin<br>M., Tu., Th., S., 1.30 to 2 p.m. |
| METROPOLITAN FREE HOSPITAL, Kingsland Road, N.<br>Admission free | Dr. Drysdale<br>„ J. G, Dudley<br>„ Fotherby<br>„ H. H. Tooth<br>„ A. Haig<br>„ A. Davies<br>Daily, 9 and 12 | Mr. E. J. Chance<br>„ Goodsall<br>„ Walsham<br>„ A.A. Bowlby<br>„ S. Paget<br>Daily, 9 a.m. |

| Hospitals and Terms of Admission. | Medical Officers, and Days and Hours of Attendance. | |
|---|---|---|
| | Physicians. | Surgeons. |
| MIDDLESEX HOSPITAL, Berners Street, W.<br>Admission free | Dr. Fowler<br>„ Biss<br>„ Pringle<br>M., Th., 1.15; Tu, F., 1.30; W., S., 9 a.m. | Mr. Andrew Clark<br>„ Pearce Gould<br>„ J. B. Sutton<br>M., Tu., Th., F., 1.30 p.m. |
| NORTH-WEST LONDON HOSPITAL, 18, Kentish Town Road, N.W.<br>By subscribers' free tickets | Dr. W. C. Hood<br>„ J. Shaw<br>„ Edwardes<br>„ Wolfenden<br>Daily, 1.30 p.m. | Mr. F. Durham<br>„ Collier<br>„ Jennings<br>„ J. Black<br>Daily, 1.30 p.m. |
| OSPEDALE ITALIANO, 41, Queen Sq., Bloomsbury.<br>Admission free to persons of all nationalities, but Italians have preference. | Dr. M. Castenada<br>„ Steggall | M. & W., 11.30 to 1; Tu., W. & F., 10 to 11 a.m.; S., 4 to 5 p.m. |
| POPLAR HOSPITAL, 303, East India Dock Road, E.<br>Free to accidents and emergencies | Dr. J. D. Grant<br>W., S., 12 to 2 p. m. | Mr. Bowkett<br>Tu., F., 12 to 2 p.m. |
| ROYAL FREE HOSPITAL, Gray's Inn Road, W.C.<br>Admission free | Dr. Cockle<br>„ Sainsbury<br>„ West<br>Daily, 1.0 | Mr. Rose<br>„ W. Gant<br>„ Brow<br>Daily, 1.0 |
| ST. BARTHOLOMEW'S HOSPITAL, Smithfield.<br>Admission free | Dr. Hensley<br>„ Brunton<br>„ Legg<br>Daily, 11 a.m. | Mr. Marsh<br>„ Butlin<br>„ Walsham<br>Daily, 12 |
| ST. GEORGE'S HOSPITAL, Hyde Pk. Corner, S.W.<br>By Governor's letter | Dr. Ewart<br>„ Owen<br>*Women*, M., Tu., 10.30; *Men*, F., S., 10.30 a.m. | Mr. Bennett<br>„ Dent<br>*Women*, M., Tu., 10.30; *Men*, F., S., 10.30 a.m. |
| ST. MARY'S HOSPITAL, Cambridge Pl., Paddington, W.<br>By Governor's letter | Dr. D. B. Lees<br>„ S. Phillips<br>„ R. Macguire<br>Daily, 1.30 | Mr. H. W. Page<br>„ W. Pye<br>„ A. J. Pepper<br>Daily, 1.30 |
| ST. THOMAS'S HOSPITAL, Westminster Bridge Road, S.E.<br>Vaccination on Wednesday at 11.30 | Dr. Payne<br>„ Sharkey<br>„ Gulliver<br>Daily, 1.30 | Mr. MacKellar<br>„ Clutton<br>„ Anderson<br>„ Pitts<br>Daily, 1.30 p.m. |
| SEAMEN'S HOSPITAL (late Dreadnought), Greenwich, S.E.<br>Entirely free to seamen, and to all accident cases | Dr. J. Curnow<br>„ H. J. Griffiths<br>Daily, 9 to 3 | Mr. W. J. Smith<br>„ G. R. Turner<br>Daily, 9 to 3 |
| TOTTENHAM TRAINING HOSPITAL, Tottenham, N. | Dr. Lloyd Smith | M., W., 11 to 2; *Men* only, W., 7 to 8 p.m. |
| UNIVERSITY COLLEGE HOSPITAL, Gower St., W.C.<br>By Governors' letters. Accidents and emergencies free | Dr. Gowers<br>„ Poore<br>„ Barlow<br>Daily, 1 30 | Mr. Barker<br>„ Godlee<br>„ Horslee<br>Daily, 1.30 p.m. |
| WEST LONDON HOSPITAL, Hammersmith Road, W.<br>By Governors' letters. New cases must attend at 1.30 | Dr. Herringham<br>„ Savill<br>„ Ball<br>Daily, 2.0 | Mr. Ballance<br>„ Weiss<br>„ Wainewright<br>Daily, 2 p.m. |
| WESTMINSTER HOSPITAL, Broad Sanctuary, S.W.<br>By Governors' letters. Accidents and urgent cases free | Dr. De H. Hall<br>„ A. H. Bennett<br>„ Murrell<br>Daily, 1.30 | Mr. Cooke<br>„ Bond<br>„ Barrow<br>Daily, 1.30 p.m. |

